# Editorial: Long-Term Consequences of Adolescent Drug Use: Evidence From Pre-clinical and Clinical Models

**DOI:** 10.3389/fnbeh.2018.00083

**Published:** 2018-05-03

**Authors:** Alonzo J. Whyte, Mary M. Torregrossa, Jacqueline M. Barker, Shannon L. Gourley

**Affiliations:** ^1^Departments of Pediatrics and Psychiatry, Yerkes National Primate Research Center, Emory University, Atlanta, GA, United States; ^2^Department of Psychiatry, Translational Neuroscience Program, University of Pittsburgh, Pittsburgh, PA, United States; ^3^Department of Pharmacology and Physiology, Drexel University College of Medicine, Philadelphia, PA, United States; ^4^Graduate Programs in Neuroscience and Molecular and Systems Pharmacology, Emory University, Atlanta, GA, United States

**Keywords:** addiction, adolescence, prefrontal, decision making, impulsivity

Adolescence is characterized by risky behavior and impulsive decision making, which often exposes adolescents to psychoactive substances. Meanwhile, neural circuits refine and mature during adolescence, creating a window of opportunity for environmental insults to impact brain maturation and potentially confer susceptibility to life-long drug addiction. Indeed, an early age of first drug use (before 15 years old) is strongly associated with risk for developing a substance use disorder later in life (Kessler et al., 2001). A large number of clinical studies have investigated both the acute and long-term effects of adolescent substance use on a number of neurobehavioral measures, resulting in a clinical research literature that can at times be conflicting, likely due to difficulties in controlling for environmental factors—including which drugs were ingested, when, and in what combinations. While animal models allow for control over all of these factors, the majority of preclinical addiction research has historically used adult animals and thus failed to address the effects of drug and alcohol exposure during adolescence. We recently organized a Special Issue of *Frontiers in Neuroscience*, meant to collect both clinical and preclinical investigations focused on factors associated with illicit drug vulnerability in adolescents, and the long-term consequences of adolescent drug exposure. This Editorial provides an overview of our Special Issue, organized in 3 themes.

## Theme 1: risks predisposing adolescents to substance use

Although many adolescents engage in risky, experimental substance use without developing later-life addiction, certain non-drug-related, functional differences appear to predispose adolescents to increased risk for addiction. Clark et al. studied a diverse and representative sample of adolescents in the National Consortium on Alcohol and Neurodevelopment in Adolescence (NCANDA) and report that poor daily executive functioning (higher-order cognitive skills such as mental flexibility that optimize responding to maximize reward) significantly relates to increased substance use risk during adolescence. Tervo-Clemmens et al. focused further on the executive function characteristics in adolescents that increase risk for substance use. In a sample of youths from the NCANDA study, Tervo-Clemmens et al. replicated previous findings indicating that poor response inhibition is associated with substance use risk. The nature of this risk was further elucidated using a reward-anti-saccade task in which participants are primed with visual cues during a fixation period. The visual cue provides information as to whether correct performance on the subsequent response trial will be rewarded (monetarily) or not incentivized. During response trials, participants must perform a saccade away from the presented visual stimulus. Although incentivizing accurate responding improved performance in the anti-saccade task, incentives alone were insufficient to modify associations between response inhibition and substance use risk, indicating that deficits in response inhibition and heightened sensitivity to reward are distinct neurocognitive features of adolescent substance use risk.

In addition to the general risk that poor daily executive function confers, early-life events and sex may also impact long-term risk for substance abuse. In their review article, Weil and Karelina summarize clinical and preclinical evidence suggesting that traumatic brain injury during adolescence increases risk for alcohol use disorder later in life. For males, this pattern is particularly pertinent, as young men are more likely to suffer from abuse-related head injury, traumatic brain injury, and also alcohol abuse. In preclinical studies, Barfield and Gourley sought to uncover sex-specific behavioral consequences of experiencing elevated levels of the primary stress hormone corticosterone during adolescence. Using an instrumental reversal learning task, Barfield reports that female, but not male, mice exhibit inflexible, perseverative-like response errors following early-adolescent corticosterone. These findings are significant because pre-existing deficits in behavioral flexibility may accelerate the transition from recreational substance use to addiction (Dalley et al., 2004; Perry and Carrol, 2008; Cervantes et al., 2013). Further, longer periods of corticosterone exposure in adolescence impair reversal performance in male mice in the same task (Shapiro et al., 2017). Related findings from Barker et al. indicate that high dose binge-like ethanol exposure during adolescence may selectively promote habitual behavior in female rats, with adolescent male rats showing no alterations in response strategy selection in adulthood. Together, these preclinical reports suggest that adolescent females are more vulnerable to developing drug- and stressor-related errors in updating action-outcome associations, which may confer vulnerability to drug use and misuse.

## Theme 2: effects of adolescent drug exposure on brain physiology and long-term behavior

Across multiple mammalian species, exposure to drugs of abuse during adolescence is associated with deficits in neurodevelopment. Using data from the longitudinal Brain and Alcohol Research in College Students (BARCS) study, Meda et al. report that, compared to the typical decline in gray matter volume associated with adolescent neurodevelopment, young adults who engage in heavy drinking display greater volume loss in regions such as the frontal cortex and hippocampus. In this investigation, participants initially underwent MRI brain scans and self-reported their drinking behavior during young adulthood (average age 18 years old) and were then sampled again 2 years later. Interestingly, Meda et al. also show that many of the participants who self-reported heavy drinking at the beginning of the study already had smaller frontal cortices, potentially suggestive of a pre-disposition toward substance abuse. Also affected by adolescent substance exposure are the white matter tracts that connect gray matter regions. Li et al. find that adolescent cynomolgus monkeys exposed repeatedly to ketamine have reduced white matter integrity in connections between brain regions involved in executive functions and learning and memory such as the frontal cortex, striatum, thalamus, and hippocampus. Neural changes are also detectable at the molecular level: Liu and Crews report that rats exposed to binge-like ethanol treatment during adolescence exhibit less neurogenesis within the dentate gyrus region of the hippocampus, and also the subventricular zone. Further, Vore et al. report that rats exposed to binge-like ethanol treatment during adolescence have reduced cytokine expression in response to stress and immune challenge.

Damage to specific neurocircuits by adolescent drug exposure is also associated with long-term effects on learning and memory and executive functions. Sanchez Marin et al. report that adolescent rats exposed to binge-like ethanol treatment not only have aberrant mRNA levels of genes encoding enzymes and receptors in the endocannabinoid system and neuroinflammation-related factors within the frontal cortex, and higher mRNA levels of stress-related genes within the hippocampus, but in addition, develop deficits in the novel object recognition test of learning and memory. Similarly, Marco et al. find that binge-like ethanol exposure not only reduces cannabinoid receptor type 2 protein levels in the frontal cortex, but also causes significant deficits in novel object recognition. Last, Kruse et al. show that, in rats, deficits in risk-based decision making (overly risky behavior), resultant from adolescent ethanol self-administration, are not caused by impulsivity but rather are due to heightened sensitivity to reward-related conditioning stimuli.

## Theme 3: preclinical studies could help to clarify cause-and-effect in risk

The nature of clinical studies often precludes direct cause-and-effect analyses of mechanisms underlying substance use risk. Several studies within this collection use preclinical models to tease out these distinctions, and they report important null and unexpected effects of adolescent drug exposure. For example, in female rats, nicotine exposure during adolescence unexpectedly fails to impair behavioral flexibility or energize ethanol self-administration and reinstatement in adulthood (Madayag et al.). Nor does adolescent ethanol exposure energize later-life self-administration in male rats (Carvajal et al.). In several psychosis-related behavioral tasks, Jaehne et al. report that, despite the known actions of methamphetamine on serotonergic neurotransmission, mice lacking the 5-HT1A receptor and exposed to methamphetamine during adolescence are indistinguishable from methamphetamine-exposed wildtype control mice. Finally, Kirschmann et al. present findings that challenge the popular belief that adolescent exposure to marijuana causes long-term deficits in executive functions. Using an operant conditioning procedure that allows adolescent rats to self-administer cannabinoids, Krishmann et al. report that adolescent cannabinoid self-administration in female rats has no detectable effects on spatial or working memory performance. Further, experimenter-administered cannabinoids actually *improved* spatial and working memory performance in adulthood.

## Summary

Together, these studies provide insight into factors that may alter adolescent brain development and increase, decrease, or have no effect on subsequent risk for addiction in adulthood. Overall, these articles highlight the necessity of clinical studies focused on the real-world risks and consequences of adolescent substance use and abuse, combined with more controlled (and controllable) preclinical studies aimed at elucidating mechanistic factors by which adolescent drug exposure impacts neurocognitive functions, neural structure, and neuronal plasticity during development and into adulthood.

## Author contributions

AW wrote the first draft of the manuscript. All other authors edited the manuscript and contributed to the final version of the manuscript. All authors approved the manuscript for publication.

### Conflict of interest statement

The authors declare that the research was conducted in the absence of any commercial or financial relationships that could be construed as a potential conflict of interest.
